# The Nature of Working Memory for Braille

**DOI:** 10.1371/journal.pone.0010833

**Published:** 2010-05-26

**Authors:** Henri Cohen, Patrice Voss, Franco Lepore, Peter Scherzer

**Affiliations:** 1 Laboratoire de Psychologie et Neuropsychologie Cognitives (FRE-3292), CNRS - Université Paris Descartes, Boulogne-Billancourt, France; 2 Institut des Sciences Cognitives, Université du Québec à Montréal, Montreal, Canada; 3 Quebec Memory and Motor Skills Disorders Research Center, Clinique Sainte-Anne, Québec, Canada; 4 Centre de Recherche en Neuropsychologie et Cognition, Université de Montréal, Montreal, Canada; University College London, United Kingdom

## Abstract

Blind individuals have been shown on multiple occasions to compensate for their loss of sight by developing exceptional abilities in their remaining senses. While most research has been focused on perceptual abilities per se in the auditory and tactile modalities, recent work has also investigated higher-order processes involving memory and language functions. Here we examined tactile working memory for Braille in two groups of visually challenged individuals (completely blind subjects, CBS; blind with residual vision, BRV). In a first experimental procedure both groups were given a Braille tactile memory span task with and without articulatory suppression, while the BRV and a sighted group performed a visual version of the task. It was shown that the Braille tactile working memory (BrWM) of CBS individuals under articulatory suppression is as efficient as that of sighted individuals' visual working memory in the same condition. Moreover, the results suggest that BrWM may be more robust in the CBS than in the BRV subjects, thus pointing to the potential role of visual experience in shaping tactile working memory. A second experiment designed to assess the nature (spatial vs. verbal) of this working memory was then carried out with two new CBS and BRV groups having to perform the Braille task concurrently with a mental arithmetic task or a mental displacement of blocks task. We show that the disruption of memory was greatest when concurrently carrying out the mental displacement of blocks, indicating that the Braille tactile subsystem of working memory is likely spatial in nature in CBS. The results also point to the multimodal nature of working memory and show how experience can shape the development of its subcomponents.

## Introduction

There has been an increasing interest in the study of blindness and its effects on cognition and behavior in recent years. An ongoing debate regarding whether blind individuals are able to compensate for their sensory handicap by developing exceptional abilities in their remaining senses [compensatory hypothesis: see 1], or by becoming severely handicapped given the importance of the visual modality in calibrating other senses [the deficit hypothesis: see 2] has yielded contrasting positions. While the results of a few studies have actually loaned support to the latter hypothesis [e.g., [3]–[5]], the vast majority of published reports either support the former hypothesis or do not show any difference between sighted and blind individuals [for reviews, see [6]–[8]]. Thus, the general view that blind individuals can exhibit enhanced abilities within their remaining senses. Although most of the early interest with blind individuals was with regards to their perceptual abilities in the auditory [9]–[12] and tactile [10], [13]–[18] domains, there has been a growing shift with respect to language [19], [20] and memory [17], [21]–[26] functions.

Although tactile and memory functions in blind individuals have received much attention, little is known about the existence of a tactile subsystem within the framework of the most generally accepted model of working memory proposed by Baddeley and Hitch [27]–[30]. Briefly, this model consists of separate but interconnected subsystems, the most important of which is the central executive, which controls processes including access to the other subsystems as well as the retrieval of information stored in long-term memory. The other subsystems or modules are essentially “slave” systems, one of which is specialized for auditory-verbal and the other for visual stimuli. The verbal system contains a phonological store where verbal input is actively represented. These representations are maintained by subvocal rehearsal in a phonological loop which, when blocked, leads to impaired recall of information. The second “slave” system, also known as the visuospatial sketch pad, is argued to be responsible for storing visuo-spatial information and creating and manipulating mental images. Though the original model only proposed components for the auditory (phonological store) and visual (visuospatial sketchpad) modalities, the results from several studies rather suggest that working memory processes extend to other sensory modalities such as touch [31]–[36]. In fact, behavioral studies have shown that visual perception is not essential for an efficient development of working memory [25], [26], suggesting that other sensory modalities might compensate by providing the necessary spatial information.

In light of the important contribution of tactile input for information processing in the blind, the purpose of this study was to further explore tactile working memory for Braille in this group of individuals via two experimental procedures. The first experiment was an immediate serial recall task with and without articulatory suppression and was carried out in order to determine how this tactile working memory in blind subjects compared to visual working memory in sighed individuals, and secondly, to determine the extent to which the nature of the visual impairment (completely blind subjects (CBS) versus blind subjects with residual vision (BRV)) had any effect on its development. The second experiment was then designed to test if this tactile component of working memory was more spatial or verbal in nature by having new groups of participants perform the task concurrently with a mental arithmetic or a mental displacement of blocks task.

## Methods

### Subjects

All participants gave written and informed consent. Subjects were excluded if they had any brain damage or other neurological illness. The twelve sighted subjects were recruited from the Université de Quebec personnel and matched as closely as possible with two visually impaired groups (CBS and BRV) with respect to age, sex and education. The CBS and BRV subjects were equivalent in Braille fluency; all had a good command of the Braille alphabet, as assessed in a pre-test, with a minimum of 14 years of practice. All visual deficits were congenital.

#### Experiment 1 (Tactile task)

Twenty-seven subjects (aged from 18 to 60 years) participated in this experiment. Fifteen subjects had a congenital visual deficiency, 7 with total blindness (CBS: completely blind subjects) and 8 with some residual vision (BRV: blind with residual vision) and 12 had normal vision (see Tables 1 and 2). The CBS subjects had a mean age of 44.14 years (ranging from 31 to 53) and the BRV subjects had a mean age of 45.63 (ranging from 41 to 53). The mean age of the control subjects was 44.6 years.The BRV group was composed of individuals who were classed as visually handicapped by the Québec Health Insurance Board, which has the legal authority in Québec to set the standards for defining a specific handicap. The definition states that a subject belonging to this category must have a visual acuity in each eye, when corrected with appropriate ophthalmic lenses not greater than 4 dioptres, of less than 6/21.

**Table 1:** Description of completely blind subjects (CBS; Experiment 1).

Subjects	Sex	Age	Education	Aetiology
1	F	47	14	Keropathy and glaucoma
2	M	31	15	Retinal detachment
3	M	48	15	Congenital cataracts and glaucoma
4	M	50	19	Retinal pigmentation
5	F	53	20	Retinal pigmentation
6	M	34	19	Congenital anophtalmia
7	F	46	18	Congenital blindness (cause unknown)

**Table 2:** Description of blind subjects with residual vision (BRV; Experiment 1).

Subjects	Sex	Age	Education	Aetiology
1	F	47	11	Congenital cataracts
2	F	41	17	Oculocutaneous albinism
3	F	51	20	Congenital cataracts
4	M	42	20	Oculocutaneous albinism
5	M	44	18	Congenital cataracts and glaucoma
6	F	45	16	Congenital cataracts
7	F	53	13	Oculocutaneous albinism
8	F	42	16	bilateral lenticular ectopia

#### Experiment 2 (Braille letter task)

Two other groups made up of nine CBS subjects with a mean age of 40.11 (ranging from 25–55 years), (see Tables 3 and 4) and 11 BRV subjects with a mean age of 48.09 (ranging from 24–58 years) participated in this experiment. These were different groups of subjects from those who participated in Experiment 1, since the ethics committees of the *Centre de Recherche Interdisciplinaire en Réadaptation*, which coordinates research with blind subjects sponsored by the *Institut Nazareth and Louis Braille*, the agency that looks after the well-being of visually handicapped individuals in Québec, does not condone the repeated use of the same subjects for research purposes. The study was also approved by the Ethics Committee of Université du Québec, where the research originated.

**Table 3:** Description of CBS (Experiment 2).

Subjects	Sex	Age	Education	Aetiology
1	F	25	18	Tapeto-retinal degeneration
2	M	29	16	Retinal nerve atrophy
3	M	52	19	Retinal pigmentation
4	M	33	16	Retinal detachment and congenital cataracts
5	F	35	18	Retinal pigmentation
6	M	50	15	Congenital cataracts and glaucoma
7	F	48	18	Congenital blindness (cause unknown)
8	F	34	14	Retinal pigmentation
9	F	55	20	Retinal pigmentation

**Table 4:** Description of BRV subjects (Experiment 2).

Subjects	Sex	Age	Education	Aetiology
1	M	52	22	Oculocutaneous albinism
2	F	51	17	Oculocutaneous albinism
3	M	47	19	Retinal nerve atrophy
4	M	53	18	Congenital cataracts
5	M	44	17	Congenital malign myopia
6	F	57	11	Congenital cataracts
7	M	58	19	Congenital cataracts
8	M	49	17	Leber's disease
9	M	37	17	Leber's disease
10	F	57	12	Congenital cataracts
11	F	24	12	Leber's Amaurosis

### Tasks

#### Experiment 1 (Tactile task)

This was an immediate serial recall task with and without articulatory suppression. The task consisted of eight series each containing two sequences of stimuli. The number of stimuli in a sequence was then increased by one with each new series (starting with two in the first series and ending with nine in the last series). The stimuli were consonants (Braille characters) presented at the rate of one per second on a Braille board. The consonants were randomly selected, and any well-known acronyms were omitted. Articulatory suppression consisted of repeating aloud the syllable/bla/during the presentation of the Braille stimuli.

A visual analogue of this tactile task was also presented to the sighted and the BRV subjects (the latter also performed the task in its tactile form). The purpose of this task was first to verify the efficacy of the articulatory suppression task in sighted subjects and to assess the visual working memory of BRV subjects. Consonants (alphabet letters) were presented at a rate of one per second on a computer screen. The order of presentation of the tasks (tactile and visual) to the BRV subjects was counterbalanced.

#### Experiment 2 (Braille letter task)

This task was a recognition task where consonants were presented at a rate of one every three seconds on a Braille board and consisted of eight series each containing three different sequences of stimuli to recall. The number of stimuli in a sequence was then increased by one with each new series (starting with two stimuli in the first series and going up to nine in the last). Again, the consonants were randomly selected, and any well-known acronyms were omitted. For each sequence, subjects were required to memorize the letters in the order in which they were presented. After a two second interval, the sequence was repeated, but this time it included a novel letter inserted into the sequence, and subjects were required to identify this new letter. This task was also carried out concurrently with a mental arithmetic task and a mental displacement of blocks task in order to determine whether the encoding of the Braille stimuli involved a spatial or a verbal strategy:

#### Mental arithmetic task

Subjects were orally presented with a series of five numbers. They were required to add these numbers, and were asked for the sum after each new number was presented. Subjects were requested to give their answers as quickly as possible. Logie, Zucco and Baddeley [37] have previously established the efficacy of this task.

#### Mental displacement of blocks task

Subjects were required to mentally represent an arrangement of three rows of two square blocks. They were presented with an initial tactile figure showing the arrangement of the stimuli. Each block was associated with a number (One through Six). The experimenter then orally specified new positions of the blocks relative to each other, and subjects were asked to mentally move the blocks into these new positions. For example, the experimenter could instruct the participants to “move Block One in front of Block Two” or “move Block Three to the right of Block Four”. The number of displacements increased in line with the number of letters in the series in the Braille letter task (for example, a sequence of three Braille letters corresponded to three block displacements). The new arrangements of the blocks resulting from the displacements of the blocks did not correspond to any Braille letters. After each new set of instructions, the subjects were asked to verbally describe the new figures (immediately after they had recalled the new letter in the Braille sequence), using the numbers corresponding to the different blocks. Subjects were told that before each new set of instructions, the blocks would start again from their initial positions.

## Results

**Figure 1:**
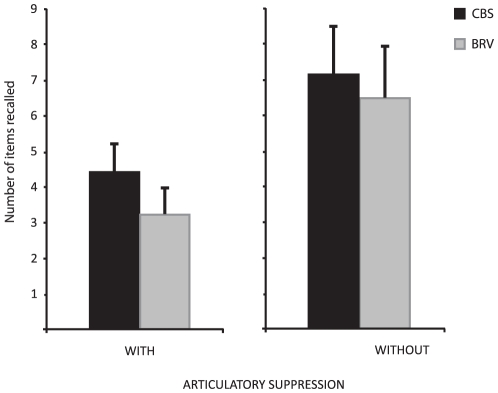
Results of the tactile task performed with and without articulatory suppression. Both groups performed similarly with no significant difference in number of items recalled when the task was performed without articulatory suppression. However, when articulatory suppression was present, the CBS group performed better than the BRV group.

The scores obtained by each subject was the maximum number of items recalled for which the subject succeeded at least once out of two tries for the first experiment, and out of three for the second. **The first experiment** was an immediate serial recall task with and without articulatory suppression. The results of CBS subjects on the Braille task were then compared to the performance of both BRV and sighted subjects on a visual version (see Methods) of the task. A two (task) by two (group) repeated measures ANOVA revealed both significant task (F = 59.68, p<0.001) and group (F = 4.57, p = 0.05) effects confirming that the task is more difficult under articulatory suppression and that the CBS showed better performances than the BRV subjects overall across both tasks. Although the group by task interaction did not reach statistical significance (F = 0.48, p>0.05), as it can clearly be seen in Figure 1, the group difference is essentially attributable to the CBS performing better than the BRV subjects under articulatory suppression, as revealed by a post-hoc contrast analysis (p = 0.009). The difference between the two groups when articulatory suppression is not present did not reach significance (p = 0.385).

**Figure 2:**
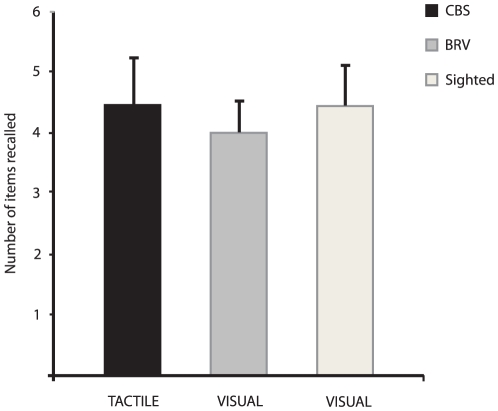
Results of the tactile task performed by the CBS subjects and of the visual task performed by BRV and sighted subjects. Under their respective conditions, all groups performed equally well.

Noteworthy is the fact that no difference was revealed when comparing tactile working memory of CBS subjects with the visual working memory of BRV and sighted control subjects under articulatory suppression (F = 1.127, p>0.05) (see Figure 2). These findings indicate that it is likely that blind subjects (CBS) have a short-term memory specific for tactile stimuli which is in effect equivalent to the short-term memory for visual stimuli possessed by subjects with partial (BRV) or full sight.

**Figure 3:**
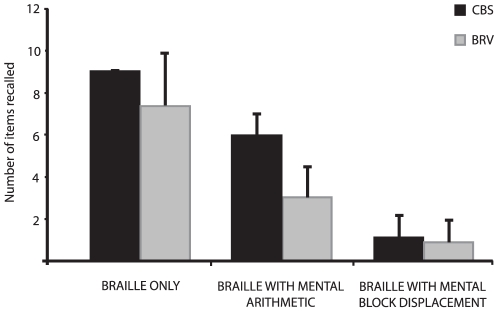
Results of the Braille task (Experiment 2). The added tasks of mental arithmetic and mental displacement of blocks significantly affected performance for both groups. Additionally, the spatial task affected significantly more than the non-spatial one, again for both groups, indicating that the tactile module of working memory is more spatial in nature. Note that the error bar is 0 for the CBS group performing the Braille task alone as all of the subjects recalled the same number of items, possibly reflecting a ceiling effect.

**The second experiment** compared the performance of a different group of CBS subjects on three different tasks to assess if the tactile module was more spatial or verbal in nature (see Figure 3). The first was a simple serial recall of Braille consonants (B). The second consisted of the same task performed concurrently with a mental arithmetic task (that is, with a verbal component- BMA) and the third task was performed concurrently with a mental displacement of blocks (that is, with a spatial component- BMDB). A two (group) by three (task) ANOVA with repeated measures on the second factor revealed significant task (F = 263.59, p<0.001) and group (F = 9.24, p = 0.007) effects as well as a task by group interaction (F = 9.40, p = 0.003). The group difference is mainly attributable to the CBS better performing the arithmetic task than the BRV subjects as demonstrated via a post-hoc contrast analysis (p<0.001). No other comparisons between the two groups reached statistical significance (p>.05) — although the two groups probably differ with regards to their ability to perform the Braille task alone, as a strong ceiling effect was observed for the CBS subjects. Further post-hoc contrast analyses revealed that all tasks were significantly different from one another for both groups (p<0.001), demonstrating not only that the mental arithmetic task and the mental displacement of blocks task significantly reduced performance compared to the Braille task alone, but also that the interference caused by the mental displacement of blocks task was significantly greater than that caused by the mental arithmetic task.

## Discussion

The principal aim of the current experiments was to investigate both the nature and the level of independence of tactile working memory for Braille in blind individuals. The first experiment was an immediate serial recall task with and without articulatory suppression and was carried out in order to determine whether blind subjects possessed a tactile subsystem of working memory for Braille and, secondly, to determine whether the type of visual impairment (completely blind subjects (CBS) versus blind subjects with residual vision (BRV)) had any effect on its development. The CBS recalled more items on average than the BRV subjects, and this was especially true when the task was designed so as to tax the short term retention of tactile Braille stimuli, suggesting that the verbal nature of the distractor affected the performance of the BRV subjects to a greater extent than that of the CBS participants.

The latter result also suggests that BrWM is more robust in individuals with complete visual deprivation than in those with partial vision. One possibility is that individuals are born with similar levels of short-term memory capacity for the different sensory modalities but, depending on sensory experience, some memory modalities become more enhanced than others. Thus, most persons have good auditory and visual short-term memory, because they have the most experience with inputs in these modalities. Consequently, the working memory subsystems might be highly crossmodal with numerous interconnections, the strengths of which are altered through experience. To test this hypothesis the results of CBS subjects on the Braille task were then compared to the performance of both BRV and sighted subjects on a visual analogue (see Methods) of the task to assess whether the tactile working memory for Braille of the blind is of comparable capacity to the visual working memory of sighted individuals. Indeed the performance of the CBS in the tactile task was found to be very similar to that of the BRV and sighted subjects in the visual task under articulatory suppression, suggesting that in the absence of visual input, tactile processes of working memory can achieve the same level of efficiency as does vision in sighted people. Moreover, these results are in strong agreement with the findings of a previous study comparing working memory for Braille and a raised-letter tactile task in blind individuals with that of visual and a raised-letter tactile in sighted individuals [22]. While the performance for the raised-letter tactile working memory task was found to be superior in the blind, the performance of the blind in the Braille working memory task was shown to be equivalent to that of the sighted performing the visual task, again strongly suggesting that tactile Braille working memory in the blind is very comparable to visual working memory in the sighted.

This result is consistent with a recent finding showing that working memory processes in both visual and tactile modalities share common neural networks [35], thus providing support for the notion of a working memory system independent of sensory modality. Additional support for the idea of a multi-modal working memory system comes from Zhou and Fuster [38], who found that during a cross-modal (visuo-haptic) delayed-matching task, neurons in the somatosensory cortex fired in response to visual stimuli which were behaviourally associated with tactile information. This could also account for Deibert et al. 's [39] findings that a tactile object recognition task elicited the participation of areas of the visual system as well as other brain areas in the somatosensory and motor systems of sighted subjects. Alternatively, one could argue that the comparable performance in both Braille and visual working memory may be a result of the use of similar verbal processes. This is an empirical question and it could be assessed in future experiments using pseudo-Braille and visual pseudo-letter stimuli.

An even more striking piece of evidence comes from studies showing that V1 [18] and the calcarine fissure [21] are activated during reading and recuperation of Braille information from memory. As well, tactile working memory tasks activate the dorsal extrastriate visual stream in congenitally blind individuals [40], perhaps explaining why tactile working memory in blind individuals achieves similar levels of efficiency as visual working memory in sighted ones. Similarly, crossmodal recruitment of V1 is also observed in sighted individuals who have been blindfolded for several days [41], a condition that has also been shown to increase tactile performance in sighted individuals [42]. Perhaps even more striking is the finding that V1 is activated when trained experts in a specific type of tactile stimuli perform discrimination tasks with such stimuli [43].

The fact that the dorsal stream was preferentially activated also suggests that tactile working memory is subserved, at least in blind individuals, by areas specialized in spatial processing. This notion is in fact strongly corroborated by the results of our second experiment. Indeed the finding that the disruption of memory was greatest when concurrently carrying out the mental displacement of blocks for both CBS and BRV subjects compared to concurrently carrying out mental arithmetic tasks, strongly suggests that the tactile subsystem of working memory used for Braille is most likely spatial in nature.

Mental rotation is a complex cognitive task recruiting several brain areas, notably BA7a,b in the parietal cortex, probably because of the task requirement of encoding spatial relations and computing movement. Area V5 is also activated, probably a reflection of the task requirement of mentalizing the rotation of the object [44]. On the other hand, mental arithmetic activates the left occipital-temporal cortex (left supramarginal sulcus, BA40), the adjoining intra-parietal gyrus (BA37/21) and the left anterior intraparietal sulcus (BA7) [45]. Thus, while it is true that both tasks have verbal components, especially since they both involve verbal instructions, there is little overlap between the neural networks involved. However, until there is a confrontation between both tasks with the same subjects, this conclusion remains to be confirmed.

Finally regarding the nature of Braille tactile working memory, it is clear that one must distinguish between verbal memory, verb generation and Braille. In the Amedi et al. [21] study, only the last condition was associated with an activation of the right occipital cortex. This region thus appears to be specific to Braille in the early blind subjects. There is clearly, in these results, a distinction between Braille memory, verbal memory and verb generation, all verbal tasks. The question is, how does the activation of this area affect the encoding of the relevant information. The results of the present study show that the encoding is spatial in character. However, it is possible that the mental rotation task may be more difficult than the mental arithmetic task, and that might explain why the former was more efficient in blocking the working memory task.

In summary, tactile working memory for Braille in the blind was found to be as efficient under articulatory suppression as was visual working memory in sighted individuals. Moreover, articulatory suppression appeared to influence the tactile working memory of CBS more than that of BRV subjects, suggesting that visual experience may play a crucial role in shaping tactile working memory. Further research is required however to verify this assumption. The results of the second experiment indicate that tactile working memory in the blind has an important spatial component, suggesting that this component may be intact in these individuals despite the absence of visual input, a spatial function that is perhaps related to an overlearned ability, that is, Braille reading.
